# What Is the Relationship between Lifestyle and Frailty Status? Data from the Portuguese Multicentre Descriptive Study

**DOI:** 10.3390/nursrep12010005

**Published:** 2022-01-28

**Authors:** Luísa Teixeira-Santos, Elzbieta Bobrowicz-Campos, Vitor Parola, Adriana Coelho, Isabel Gil, Maria de Lurdes Almeida, João Luís Apóstolo

**Affiliations:** 1 Center for Health Technology and Services Research, 4200-450 Porto, Portugal; 2 Centre of 20th Century Interdisciplinary Studies, Faculty of Psychology and Educational Sciences, University of Coimbra, 3000-115 Coimbra, Portugal; elzbieta@fpce.up.pt; 3 Health Sciences Research Unit, Nursing, Nursing School of Coimbra, 3000-232 Coimbra, Portugal; vitorparola@esenfc.pt (V.P.); adriananevescoelho@esenfc.pt (A.C.); igil@esenfc.pt (I.G.); mlurdes@esenfc.pt (M.d.L.A.); apostolo@esenfc.pt (J.L.A.)

**Keywords:** older adults, frailty, circadian health, lifestyle, citizen engagement

## Abstract

This observational and descriptive study attempted, within the scope of the ModulEn Research Project, to determine associations between lifestyle-related variables and frailty involving 160 community-dwelling older adults aged between 65 and 80 years living in the Central Region of Portugal. Forty-three percent of the study participants were pre-frail and 18% were frail. More than 50% of the frail people had slight cognitive decline, and the frailty condition was more frequently observed in women. As the literature highlights, there is potential for greater reversibility in the pre-frailty condition. To contribute to this reversibility, it is necessary to resort to interventions that promote physical activity and cognitive stimulation, apply adequate eating habits, and/or encourage the adoption of an active and socially integrated lifestyle. A healthy lifestyle implies good sleep and eating habits, and correct metabolic control that allows for effective surveillance of dyslipidemia, diabetes, and blood pressure.

## 1. Introduction

Ageing does not inevitably lead to frailty; however, frailty usually intensifies with advancing age [1,2,3]. Other determinants of frailty have also been identified, including genetics, consequences of acute or traumatic pathologies, and unhealthy lifestyles [4,5]. These determinants can be potentiated by inflammatory and immunological mechanisms, leading to the appearance of typical frailty symptoms, with subsequent adverse results, such as falls, disability, and dependence [6].

Frailty can be described as a clinical syndrome of a multifactorial nature that is characterized by a state of physiological vulnerability resulting from decreased energy reserves and reduced resistance to stressors, with a consequent decline in physiological systems [7]. Frailty can be reversed. In the pre-frailty states, there is greater potential for reversibility. With the increase of disability/dependency, this potential decreases. The prevalence of frailty tends to increase in individuals with a lower socioeconomic level, with more comorbidities and with an increased need for long-term care or additional support [5].

One of the possible consequences of ageing is physical impairment, often responsible for increased sedentary behaviour and social isolation of older people, which, in turn, contribute to decreases in their physical capacity and to cognitive decline that does not depend on a neurodegenerative condition [8]. Regarding physical activity, the Portuguese are not very active. Only 57.1% of the adult population has moderate weekly physical activity, slightly above Italy and Spain, with the average value of OECD countries being 66.5%. Physical inactivity and dietary patterns directly contribute to the percentage of the adult population with overweight or self-reported obesity [9].

Sleep disturbances are another common problem in the older population, frequently associated with adverse outcomes, such as obesity, hypertension, cognitive decline, depression, and death [10]. Older people who sleep five or fewer hours a night had an increased risk of suffering from frailty compared to those who slept seven to eight hours. On the other hand, sleep duration equal to or greater than nine hours was associated with an increased risk of pre-frailty/frailty, and the correlations seem not to be affected by other factors, such as sleep quality. Daytime sleep disorders are also significantly related to the pre-frailty and frailty conditions [11].

According to the annual report of the Portuguese Observatory of Health Systems [12], Portuguese people are increasing their consumption of antidepressants and anxiolytics, specifically older people who are mostly polymedicated (59.2%).

Eating patterns also seem to correlate with the frailty states. Especially in industrialized countries, body weight and body mass index (BMI) are increasing in people aged between 60 and 80 years [13]. Several studies have examined the association between eating patterns and frailty, showing that a diet rich in fruits, vegetables, and whole grains may be related to a reduced risk of frailty [14]. An older person’s nutritional status, as well as body composition, may also influence their frailty condition. A Portuguese study [15] found significant associations between frailty and weight; however, these associations were not confirmed in terms of BMI. In advanced age, it is also important to avoid malnutrition, protein deficiency, and weight loss [5], as the frailty/BMI association seems to develop in two ways—namely, low weight and sarcopenia predisposes one to the greatest vulnerability, and obesity predisposes one to the greatest risk of comorbidity and functional disability.

In Portugal, studies in this field are scarce. A Portuguese pioneering investigation [16] focused on testing the frailty phenotype in a population residing in the community that showed a high prevalence of frailty (34.9%), highlighting that it is more frequent in women and at older ages. It also identified low physical activity and slowness as the most frequent frailty criteria. In sociodemographic terms, frail older people were widowed/separated/divorced (46.7%) and illiterate (71.1%), lived within a family with some dependency (41.6%) and in inadequate conditions (44.9%), maintained restricted social relationships (54.2%), and had no social support (37.6%).

In another Portuguese study [14], which focused on frailty and associated risk factors in independent older people living in rural areas, almost half of the older people had pre-frailty, and the severity levels of frailty increased significantly with advancing age. According to this study, the profile of a frailty person is marked by the presence of poor self-perception of health status, greater intensity of self-reported pain, sensory problems, and use of gait aids.

Early recognition of frailty symptoms is essential to avoid or, at least, reduce the predominant implications for the morbidity and mortality of older people. There is a growing need to implement and evaluate new solutions that allow for early detection of this condition and that can guarantee an efficient and sustainable response from health systems to intervene accordingly. Given this, this study aims to determine associations between lifestyle-related variables and frailty involved.

## 2. Materials and Methods

### 2.1. Study Design

This investigation was conducted within the framework of the ModulEn Study [17] aiming to develop and validate a predictive model for frailty. It was designed as a multicentred observational descriptive study, being carried out in the Central Region of Portugal between October 2018 and October 2019.

The study was approved by the Ethics Committee of the Health Sciences Research Unit: Nursing of Nursing School of Coimbra (Opinion no.510/06-2018). The study procedures follow the Declaration of Helsinki and with the General Data Protection Regulation. All participants volunteered to participate and gave their written consent to be a part of this study.

### 2.2. Study Sample

The study participants were older adults living in the community, aged 65–80 years, and recruited by family nurses in cultural and sports associations, municipal services, and day centres. The main criteria for inclusion were the absence of moderate to severe cognitive decline and the absence of an unstable clinical condition.

### 2.3. Materials and Procedures

All eligible participants were asked to provide sociodemographic (gender, age, years of formal education, marital status), anthropometric (weight, height) and clinical (chronical conditions and intake medication) data. Then, they were asked to complete questionnaires about their sleeping and eating habits and perform tasks to identify physical and cognitive frailty symptoms.

Physical frailty was diagnosed based on the criteria of Fried’s phenotypic model [3] and following the assessment procedures defined by the authors of the Portuguese version of the test [18]. Data on fatigue were collected through a two-item questionnaire. For a physical activity assessment, a short version of the International Physical Activity Questionnaire (IPAQ) [19], developed for older adults, was used. The remaining components were assessed through objective measures. Participants who had three to five symptoms were considered frail, and those who had one or two symptoms as pre-frail. The absence of symptoms indicated a robust health status.

Cognitive frailty-related data was collected using the Portuguese version [20] of the Six-Item Cognitive Impairment Test (6-CIT) [21] that allows to screen the presence of changes in orientation in time and space, working memory/attention, and verbal memory. The cut-off points for cognitive impairment depend on the years of formal education completed by the person assessed [20].

Sleeping habits were evaluated based on the Epworth Sleepiness Scale (ESS) [22] and the Pittsburgh Sleep Quality Index (PSQI) [23]. The ESS is a self-administrated questionnaire measuring daytime sleepiness and the likelihood of falling asleep in particular situations. The PSQI assesses sleep quality and sleep disturbance over the last month.

Eating habit-related data were collected through a questionnaire on types and times of meals taken on workdays and days off.

### 2.4. Statistical Analysis

Categorical variables were analysed based on chi-square (χ^2^) tests. To identify the strength of associations, Cramer’s *V* coefficient was calculated. As for continuous variables, comparative analyses were performed using the Kruskal-Wallis test as appropriate. If differences were statistically significant, a multiple comparison of mean ranks was performed. The effect size was determined by coefficient partial eta-squared (η^2^_p_). To identify the magnitude of the relationships between continuous variables, Spearman correlations (*rho*) were calculated. The threshold for statistical significance was set at 0.05. Data were analysed using the SPSS Statics (v.24, IBM SPSS, New York, NY, USA) program.

## 3. Results

### 3.1. Demographic Data

The participants’ characteristics are presented in Table 1. The sample included 160 older people, of which 120 were female. Fifty-two percent of the sample were married and 94% were retired. The mean age was 71.77 ± 4.94 years. Regarding formal education, the participants completed, on average, 7.79 ± 4.12 years of school (range from 0 to 13).

### 3.2. Clinical Data

According to the phenotypic model of diagnostic criteria [3], 39% of participants were robust, 43% were pre-frail, and 18% were frail. Reduced grip strength and reduced activity were the most common symptoms among the non-robust (meaning pre-frail and frail) participants (Table 2). Further analysis of the IPAQ data, focused on sedentary behaviours, showed that such behaviours were common in the entire study sample (51% of all participants), their presence being confirmed in 34% of robust participants, 49% of pre-frail participants, and 90% of frail participants.

Weight loss was the symptom reported less often. Indeed, data collected on the BMI showed that study participants, either robust or non-robust, were, on average, overweight. The highest indexes of the BMI were observed in the group of frail participants. Between-group differences, analysed with the Kruskal-Wallis test, were statistically significant (*p* = 0.034). However, the multiple comparison of mean ranks revealed that pairwise differences were not significant.

The presence of mild changes in cognitive functioning, evaluated using the 6-CIT, was verified in 18% of robust participants, 20% of pre-frail participants, and 52% of frail participants. The 6-CIT score and the number of physical frailty symptoms was significantly correlated. The correlation magnitude was weak (rho = 0.159; *p* = 0.044).

Multimorbidity was reported in 92% of the cases (Table 1). Differences between the groups, analysed with the Kruskal-Wallis test, were only marginally significant. Hypertension, diseases of the senses, and musculoskeletal and connective tissue disorders were the conditions most frequently reported in the study sample (54%, 46%, and 42%, respectively). Data for each group are displayed in Table 3.

Eighty-three percent of the study participants were medicated (Table 1). The Kruskal-Wallis test showed that the between-group differences are statistically significant. The multiple comparison of mean ranks confirmed the presence of significant differences between the frail and robust older adults (*p* < 0.01) and the frail and pre-frail older adults (*p* < 0.05). The effect size was medium (η^2^_p_ = 0.074). The most common types of drugs taken by the participants were antihypertensives (administrated to 60% of the sample), anxiolytics (reported by 44% of the sample), and beta-blockers (used by 29% of the sample). Table 4 presents data for each group.

Both the number of chronic conditions and number of types of medication taken correlated significantly with the BMI (rho = 0.231; *p* = 0.003 and rho = 0.184; *p* = 0.020, respectively). The correlations were of weak magnitude.

### 3.3. Sleep and Food Habits-Related Data

In all three groups, most participants showed patterns of normal sleepiness, but had poor quality of sleep (Table 5). Between-group differences on the ESS- and PSQI-related variables, analysed using the chi-square test, were not significant. The lack of significant differences was also revealed for the ESS and PSQI total score, analysed based on the Kruskal-Wallis test. As for the score obtained in different PSQI subscales, a statistically significant difference was found for sleep disturbance (*p* < 0.001). The pairwise comparison of mean ranks revealed that the frail participants had less sleep disturbance when compared to the robust and pre-frail participants (for both: *p* < 0.001; η^2^_p_ = 0.232).

Regarding data on food habits (Table 6), all participants reported having breakfast, lunch, and dinner regularly, both on working days and days off. A second breakfast, an afternoon snack, and supper were taken by only some of the participants. There were no significant differences between the groups in the number of meals taken per day.

## 4. Discussion

Taking into account the diagnostic criteria defined in Fried’s phenotypic model [3], pre-frailty and frailty are prevalent in Portuguese older adults. In the present study, the percentage of older people with frailty was higher as compared to other studies in the same field that obtained samples with 4% to 7% of frail individuals [3,24,25]. Additionally, a systematic review that pooled findings from 61,500 older community-dwelling participants revealed that, on average, 10.7% of adults aged 65 years or more were frail, and another 41.6% were pre-frail [2], which, concerning frailty, remains below the values we observed. However, it is important to note that in Europe, southern European countries have the highest prevalence of frailty and pre-frailty. From this perspective, the percentage of frail older people obtained in the present study (18%) is lower than the rate achieved in Spain (27.3%) and Italy (23%) [1]. The percentage obtained in the present study is still consistent with another study recently carried out in Portugal (21.5%) [26].

It is accepted that frailty is centered on an ageing mechanism. However, frailty is not an inevitable consequence of the aging process [27], and age per se is of little value for discriminating between the robust, prefrail, and frail in the community [28]. Nonetheless, although there is no statistically significant difference in age between the groups, we cannot disregard its clinical relevance, since the presence of older age was observed in the frail group of older adults.

It is also important to note that the frailty assessment instruments can provide different data regarding the prevalence of frailty [28], so it would be interesting to replicate the present study using another assessment tool. In any case, the data obtained must be considered in the planning and implementation of the geriatric health policy.

Concerning gender, the data obtained confirm the existing evidence, showing that the prevalence of frailty is higher in women than in men [2,3,26,29,30,31].

The present study found that reduced grip strength was the most common symptom among the non-robust. These results are corroborated by other studies [26,31,32] which suggested that weakness develops first, it being the most prevalent phenotypic criteria.

Sedentary behaviours are related to adverse health outcomes in older adults. Our study showed that such behaviours were quite common in the entire study sample (51% of all participants), their presence being confirmed in 34% of robust older adults, 49% of pre-frail older adults, and 90% of frail older adults. These data are also relevant since, in our study, the second most common frail symptom was reduced activity. According to the literature, sedentary time is associated with higher mortality among people who are vulnerable, frail, or physically inactive [33,34]. Moreover, low physical activity is one of the most powerful predictors of future disability in activities of daily living [35].

On the other hand, similarly to other studies (1,6), weight loss was the least frequently reported symptom. In fact, the data collected at the BMI revealed that the study participants were, on average, overweight. The highest BMI indexes were observed in the group of frail participants. This could be explained by the fact that our study showed that sedentary behaviours were quite common in the entire study sample (mostly in the frail group), adding to the fact that the second most common frail symptom was reduced activity. As mentioned in the literature, sedentary behaviour increases the risk of overweight [36]. The existence of a significant association between frailty/pre-frailty and overweight has also been reported in other studies [37,38].

In opposition to these results, in a cross-sectional cohort study conducted with older Japanese citizens from a community setting, high prevalence ratios of anorexia of aging among frail older adults were found [36]. Further studies are needed to identify the determinants of both obesity and anorexia in frail older people and to define an action plan for the minimization of their impact.

Although we know that food is associated with positive health outcomes across the life-span, the relationship with health benefits among older adults has received little attention [37]. The present study did not reveal significant differences between the groups in the number of meals taken per day. These results are corroborated by another study that mentions that meal frequency does not predict the development of frailty in older adults [36]. Despite the above, it is known that high BMI is a risk factor for the development of comorbidities and the consumption of medication, and those, in turn, are important determinants of frailty [5,38].

As for medication, present results show statistically significant differences regarding the quantity of medication taken, with frail participants being more medicated than pre-frail or robust older adults. These results are in line with what was observed in another study [39]. However, a review carried out by Fenge et al. [29], analysing medication use, reported no significant association between this variable and frailty.

The present study results reveal that the presence of mild changes in cognitive functioning was verified in 18% of robust older adults, 20% of pre-frail older adults, and 52% of frail older adults, and the 6-CIT score and the number of physical frailty symptoms was significantly correlated. These results are in line with other studies’ findings that showed an association between physical frailty and poorer cognitive function [40,41]. Indeed, cognitive frailty is a heterogeneous clinical manifestation branded by the concurrent occurrence of physical frailty and cognitive impairment [42].

Poor sleep quality is prevalent among older people and compounded by frailty [11,43,44]. However, our data revealed that the frail participants had significantly less sleep disturbance when compared to the robust and pre-frail participants. This contrasts with data from the existing literature, where an association of sleep disturbance with frailty and pre-frailty was observed [11]. Perhaps the data obtained in the present study may be justified by the high percentage of frail older people who took anxiolytics, compared to pre-frail or robust older people.

Regarding limitations, as our sample was very small, there was no option to check for combined effects within the sample. In the future, we intend to carry out a cohort study to identify the changes over time in frail, pre-frail, and robust older adults (with a more representative sample) to understand how lifestyle changes frailty status.

The data presented have the potential to lead to better-quality decision-making and management of care by considering older adults’ vulnerabilities and propensity for adverse health outcomes. To successfully prevent or manage frailty, we must appreciate its multifaceted processes, many of which have not yet been evidently identified. This study is novel and has some strengths, since it presents a multidimensional and integrative view of different components related to frailty (physical, cognitive), discussing the relationship of frailty with clinical and neuropsychological characteristics, chronic conditions, medication taken, and sleep and food habits, thus allowing a comprehensive view of ageing processes.

## 5. Conclusions

In the studied population, the percentage of frail and pre-frail participants was high (18% and 43%, respectively). The study results suggest that the profile of frail older people is marked mainly by the reduction of grip strength and low physical activity, associated with sedentary behaviours. Frail participants also presented with the highest BMI values. Moreover, frail people reported, on average, six chronic health conditions, specifically and in order of prevalence, arterial hypertension, anxiety, sensory deficits, cardiovascular diseases, musculoskeletal disorders, and mental/behavioural disorders. They were also polymedicated, with the most consumed drugs including antihypertensive and anxiolytic drugs and beta-blockers. Finally, more than 50% of the frail people had slight cognitive decline.

These findings reinforce the need for considerable investment in the prevention and/or management of the condition of frailty. This investment must contemplate multiple domains, focusing on the physical, nutritional, cognitive, and psychological dimensions of individual functioning. The early implementation of suitable interventions is also suggested, since the potential for reversibility is greater in one’s pre-frail status.

## Figures and Tables

**Table 1:** Demographic, clinical, and neuropsychological characteristics of study participants.

	Robust Older Adults (*n* = 62)	Pre-Frail Older Adults (*n* = 69)	Frail Older Adults (*n* = 29)		
	%	%	%	Χ^2^	*V* de Cramer
Gender: Female/Male	69/31	80/20	79/21	2.161	0.339
Marital Status: single/married/widowed/ divorced	5/59.5/ 20.5/15	3/52/ 36/9	7/45/34/14	5.822	0.443
	Mean (SD)	Mean (SD)	Mean (SD)	Kruskal-Wallis (*p*)	multiple comparison of mean ranks
Age	71.79 (4.83)	71.77 (5.25)	73.11 (4.74)	0.255	--------
Education	8.03 (4.01)	8.12 (4.14)	6.52 (4.20)	0.146	--------
Body mass index	26.56 (3.97)	26.80 (4.20)	29.99 (6.47)	0.034	--------
Types of medication taken	1.5 (1.28)	1.75 (1.32)	2.52 (1.33)	0.003	R ^1^, P ^2^ < F
Comorbidities	4.79 (2.35)	4.88 (2.60)	6.10 (2.35)	0.059	--------

F: frail older dults; P: pre-frail older adults; R: robust older adults ^1^ *p* < 0.01; η^2^_p_ = 0.232; ^2^ *p* < 0.05; η^2^_p_ = 0.232.

**Table 2:** Percentage of older adults with symptoms of cognitive and physical frailty.

			Non-Robust Older Adults		
Presence of Symptoms		Robust Older Adults (*n* = 62)	Total (*n* = 98)	Pre-Frail Older Adults (*n* = 69)	Frail Older Adults (*n* = 29)
Physical frailty ^1^	Weight loss	-------	2.0%	0%	6.9%
	Fatigue	-------	28.6%	10.1%	72.4%
	Reduced Activity	-------	50.0%	36.2%	82.8%
	Reduced speed	-------	39.8%	18.8%	89.7%
	Reduced grip strength	-------	72.4%	63.8%	93.1%
Cognitive frailty ^2^	Mild cognitive impairment	17.7%	29.6%	20.3%	51.7%

^1^ Based on the diagnostic criteria defined in Fried’s phenotypic model. ^2^ Based on the score obtained in the six-item Cognitive Impairment Test.

**Table 3:** Percentage of older adults with certain chronic conditions.

	Robust Older Adults (*n* = 62)	Pre-Frail Older Adults (*n* = 69)	Frail Older Adults (*n* = 29)
Neoplasms	4.8%	4.4%	3.5%
Blood and immune system disorders	11.3%	15.9%	6.9%
Endocrine and metabolic diseases	6.5%	14.5%	10.3%
Mental and behavioural disorders	22.6%	27.5%	17.2%
Central Nervous System Diseases	0.00%	1.5%	3.5%
Diseases of the senses	**48.4%**	**53.6%**	**24.1%**
Cardiovascular diseases	35.5%	40.6%	20.7%
Respiratory system diseases	9.7%	17.4%	10.3%
Digestive diseases	22.6%	20.3%	10.3%
Skin diseases	11.3%	10.1%	3.5%
Musculoskeletal and connective tissue disorders	**45.2%**	**47.8%**	20.7%
Genitourinary Disorders	11.3%	8.7%	3.5%
Hyperthyroidism	3.2%	4.4%	6.9%
Hypothyroidism	6.5%	8.7%	3.5%
Hypertension	**54.9%**	**47.8%**	**65.5%**
Restless legs syndrome	3.2%	0.0%	3.5%
Narcolepsy	0.00%	0.0%	0.00%
Obstructive sleep apnea	11.3%	13.0%	13.8%
Anxiety	27.4%	31.9%	**37.9%**

**In bold:** values of the most frequent comorbid conditions.

**Table 4:** Percentage of older adults who, in the past 30 days, took certain types of medication.

	Robust Older Adults (*n* = 62)	Pre-Frail Older Adults (*n* = 69)	Frail Older Adults (*n* = 29)
Anxiolytics	40.3%	40.6%	62.1%
Antihypertensive	58.1%	56.5%	72.4%
Beta-blockers	21.0%	30.4%	44.8%
Hypnotics	13.0%	17.4%	27.6%
Corticosteroids	3.2%	10.1%	13.8%
Anti-inflammatories	1.6%	1.5%	6.9%
Melatonin receptor agonists	0.00%	0.0%	0.0%
Thyroid hormones	9.7%	15.9%	13.8%
Muscle relaxants	1.6%	1.5%	3.5%
Antipyretics	1.6%	1.5%	3.5%
Melatonin	0.0%	0.0%	3.5%

**Table 5:** Group differences observed in the Epworth Sleepiness Scale and Pittsburgh Sleep Quality Index.

	Robust Older Adults (*n* = 62)	Pre-Frail Older Adults (*n* = 69)	Frail Older Adults (*n* = 29)		
	%	%	%	Χ^2^	*V* de Cramer
Epworth Sleepiness Scale: excessive/normal sleepiness	27/73	39/61	41/59	2.602	0.272
Pittsburgh Sleep Quality Index: bad/good sleep quality	93.5/6.5	88/12	76/24	5.915	0.052
	Mean ± SD (range)	Mean ± SD (range)	Mean ± SD (range)	Kruskal-Wallis (*p*)	multiple comparison of mean ranks
Epworth Sleepiness Scale	5.45 ± 3.97 (0–15)	6.70 ± 4.54 (0–18)	6.48 ± 5.80 (0–21)	0.333	--------
Pittsburgh Sleep Quality Index	8.68 ± 3.24 (2–17)	8.51 ± 3.8 (0–17)	6.79 ± 3.22 (1–12)	0.067	--------
(a) subjective sleep quality	1.21 ± 0.55	1.20 ± 0.72	0.93 ± 0.70	0.161	--------
(b) sleep latency	1.03 ± 1.16	1.10 ± 1.06	0.90 ± 0.94	0.695	--------
(c) sleep duration	1.05 ± 0.88	1.04 ± 0.76	0.97 ± 0.68	0.898	--------
(d) habitual sleep efficiency	2.21 ± 1.04	2.17 ± 1.06	2.03 ± 1.09	0.679	--------
(e) sleep disturbance	0.98 ± 0.38	0.68 ± 0.50	0.52 ± 0.57	<0.001	P ^1^, R ^1^ > F
(f) use of sleeping medication	1.61 ± 1.50	1.45 ± 1.47	1.00 ± 1.39	0.207	--------
(g) daytime dysfunction	0.58 ± 0.98	0.86 ± 1.22	0.45 ± 0.69	0.479	--------

F: frail older adults; P: pre-frail older adults; R: robust older adults; ^1^ *p* < 0.001; η^2^_p_ =0.232.

**Table 6:** Percentage of persons taking daily meals.

Non-Robust Older Adults					
Meals	Robust Older Adults (*n* = 62)	Pre-Frail Older Adults (*n* = 69)	Frail Older Adults (*n* = 29)	Kruskal-Wallis (*p*)	Multiple Comparison of Mean Ranks
working day meals (Mean ± SD; (range))	4.87 ± 0.82 (3–6)	4.65 ± 0.97 (3–6)	5.07 ± 0.92 (3–6)	0.087	-
day off meals (Mean ± SD; (range))	4.79 ± 0.87 (3–6)	4.61 ± 0.96 (3–6)	4.90 ± 1.08 (3–6)	0.270	-
working days breakfast (% of taking meal)	100%	100%	100%		
day off breakfast (% of taking meal)	100%	100%	100%		
working days second breakfast (% of taking meal)	53.2%	52.2%	62.1%		
day off second breakfast (% of taking meal)	50.0%	49.3%	51.7%		
working days lunch (% of taking meal)	100%	100%	100%		
day off lunch (% of taking meal)	100%	100%	100%		
working days afternoon snack (% of taking meal)	82.3%	72.5%	86.2%		
day off afternoon snack (% of taking meal)	77.4%	73.9%	79.3%		
working days dinner (% of taking meal)	100%	100%	100%		
day off dinner (% of taking meal)	100%	100%	100%		
working days supper (% of taking meal)	51.6%	40.6%	58.6%		
day off’ supper (% of taking meal)	51.6%	37.7%	58.6%		

## Data Availability

The data presented in this study are available on request from the corresponding author. The data are not publicly available due to the privacy term signed by the participants in the informed consent.
